# The Use of Cocaine by Hypodermic Injection for Extraction of Teeth

**Published:** 1885-06

**Authors:** D. W. Barker

**Affiliations:** Brooklyn, N. Y.


### THE USE OF COCAINE BY HYPODERMIC INJECTION FOR EXTRACTION OF TEETH.

BY D. W. BARKER, M. D. S., BROOKLYN, N. Y.

The results of the use of Cocaine in dental practice would seem to indicate that its principal usefulness is to be found in surgical, rather than operative work; that its great power of temporarily suspending the sensation of pain is especially useful in that most painful operation which our patients undergo at our hands—the extraction of teeth. The injection of the solution over the track of the inferior dental nerve was a partial failure, because the drug is not diffusive in its operation, but decidedly local. Bathing the solution on the gum may produce a superficial insensibility, but the pain caused by extracting a tooth comes from a deeper source; hence the necessity for getting the agent deeply into the tissues. In the majority of cases, complete insensibility to the pain of extraction may be produced by injecting, with an ordinary hypodermic syringe, five drops of a four per cent solution under the gum, directly over the root of the tooth to be extracted. It will take from five to eight or nine minutes to get the full anæsthetic effect of the Cocaine. Pricking the gum to test its insensibility will indicate when to extract. The extent of insensibility is limited to a small space around the place of injection, and the effect lasts from ten to fifteen minutes, and then passes away.

An important fact to be kept in mind is that the agent is injected directly into the circulation of the patient; hence the drug and its solution should be the purest possible, and the instrument absolutely clean. In order that the solution may be of the best, it should be made of distilled water; all ordinary water contains some organic matter, and a solution made of it will sour in about a week, and become not only unfit for use, but positively dangerous. If distilled water cannot be obtained, then ordinary water that has been boiled may be employed, but a solution made from it should not be used if it has been made for more than a week. Of the various manufactures of Cocaine I prefer that made by Dr. Squibb, of Brooklyn, because of its absolute purity; the crystals are pure white, and the solution colorless.

Extreme care should be taken to keep the syringe and its point absolutely clean, and it should never be used for any other purpose; each time, after it is used, it should be cleaned and dried, by drawing a small quantity of alcohol through it. In making the injection care must be taken to inject no air into the tissues; this may be avoided by drawing some of the solution into the syringe, then turning the point upward and expelling the liquid; any air in the syringe will go out first; then fill the syringe as full as required. To avoid running the point against the edge of the alveolus, and also to avoid the thick and somewhat tough margin of the gum, let the point enter the gum an eighth of an inch from the margin, and, following the surface of the bone, pass in at least three-eighths of an inch; if the beveled side of the point is held next the bone, it will avoid sticking against it. Press on the piston rod gently and slowly, so as to expel the liquid a drop at a time; inject half of the five drops on one side (buccal or labial) of the tooth, and the remaining half on the other (palatine or lingual) side; hold the syringe point still for half a minute, and then withdraw it slowly, so that the liquid may be taken up by the tissues and not spurt back when the point is withdrawn. For molars, injection may best be made horizontally, instead of vertically. If there are two or three roots standing close together, the injection should be made midway between them. The power of feeling is not wholly suspended, for the patient can feel the lance pass around a root, and feel the tooth leave the socket, but cannot feel the pain. Teeth which have live pulps, when leaving the socket, sometimes produce a sharper sensation than those without, caused probably when the nerve is severed at the apical foramen; but this feeling is seldom described by the patient as pain; by some it is said to be like the twinge felt when one's foot gets "asleep," and some deny feeling it at all. If pain is felt, it will probably be because the operator has not waited long enough after injecting the Cocaine, or he has not made the application properly.

I have been asked if the injection itself does not cause pain. If the gum is first bathed with the solution and the syringe needle is kept very sharp, there need be little cause for complaint. The wound always heals kindly and quickly, and there is never any swelling or pain, or any sign of local inflammation or systemic disturbance; the place where the injection is made causes no trouble, but heals with the rest of the wound, and all sign of it disappears.

